# Erratum to: Controlling the plasmon resonance via epsilon-near-zero multilayer metamaterials

**DOI:** 10.1515/nanoph-2022-0372

**Published:** 2022-07-11

**Authors:** Mohsin Habib, Daria Briukhanova, Nekhel Das, Bilge Can Yildiz, Humeyra Caglayan

**Affiliations:** Faculty of Engineering and Natural Sciences, Photonics, Tampere University, 33720 Tampere, Finland

After the publication of this article, the authors found that Figure 2d is the same as Figure 3d and hereby correct Figure 2 as:

**Figure 2:**
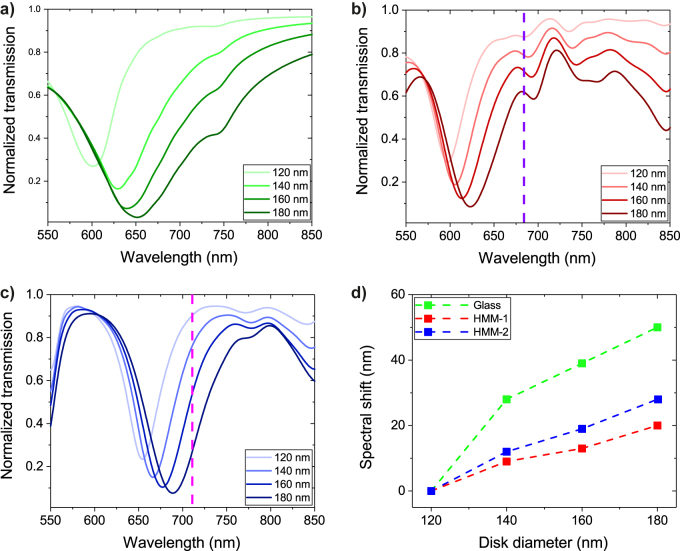
Evolution of the plasmon resonance with varying diameters of NDs on (a) glass (b) HMM-1, (c) HMM-2, and (d) spectral shift of the resonance, calculated by FDTD simulations. FDTD, finite-difference time-domain; HMM, hyperbolic metamaterial; ND, nanodisk.

